# Efficacy of epidermal growth factor receptor (EGFR)-tyrosine kinase inhibitors (TKIs) in targeted therapy of lung squamous cell carcinoma patients with EGFR mutation: a pooled analysis

**DOI:** 10.18632/oncotarget.15726

**Published:** 2017-02-25

**Authors:** Jingqi Zhuang, Yongfeng Yu, Ziming Li, Shun Lu

**Affiliations:** ^1^ Department of Oncology, Shanghai Chest Hospital, Shanghai JiaoTong University, Shanghai, People’s Republic of China

**Keywords:** efficacy, lung squamous cell carcinoma, epidermal growth factor receptor mutation, tyrosine kinase inhibitors, pooled analysis

## Abstract

**Purpose:**

This pooled analysis aims to evaluate the efficacy of epidermal growth factor receptor (EGFR)-tyrosine kinase inhibitors (TKIs) in lung squamous cell carcinoma with EGFR mutation.

**Methods:**

Advanced stage (IIIB/IV) lung squamous cell carcinoma patients with EGFR mutations treated with EGFR-TKIs were extracted from the publications searched from the databases of EMBASE, Medline (Ovid SP), Web of Science, Cochrane library, PubMed Publisher, ASCO meeting abstract and Google Scholar before August 2016, or identified from the database of Shanghai Chest Hospital from July 2014 to August 2016. Pooled objective response rate, disease control rate and median progression-free survival were accessed directly or by Kaplan-Meier method and combined in different studies by Comprehensive Meta Analysis software *via* one-group dichotomous or continuous analysis functions.

**Results:**

The combined objective response rate, disease control rate and median progression-free survival were 31.6% (95%CI, 24.1%∼40.2%), 72.0% (95% CI, 63.5%∼79.2%) and 3.08 months (95% CI, 2.31-3.84 months) in lung squamous cell carcinoma patients with EGFR mutation.

**Conclusion:**

The EGFR-TKIs had a modest response for EGFR mutated lung squamous cell carcinoma patients and might be a selective option for those patients.

## INTRODUCTION

Squamous cell lung cancer accounts for about 25–30% of NSCLC.[1] Progress in the management of advanced lung squamous cell carcinoma (LSCC) has been lagged behind.[2] For example, pemetrexed monotherapy and platinum-based doublet chemotherapy was not approved in patients with squamous histology because of inferior efficacy.[3] Nonetheless, bevacizumab, the vascular endothelial growth factor (VEGF) inhibitor, was contraindicated in LSCC due to pulmonary hemorrhage [4–6].

In the last decade, the FDA had approved targeted agents as initial treatment for patients with NSCLC, including gefitinib, erlotinib, and afatinib for the patients with EGFR mutations. Recent series including the Lux-Lung 8, [7] a meta-analysis [8] and a review [9] had shown that EGFR-TKIs had a modest therapeutic effect in unselected patients with advanced LSCC, strengthening the potential efficacy of EGFR targeted therapy in EGFR mutated LSCC. Even it has been proved that EGFR-TKIs had a better response in EGFR mutation selected lung adenocarcinoma, whether the EGFR-mutated LSCC patients can benefit more from the EGFR-TKIs remains unclear. However, former pooled frequency of LSCC patients with EGFR mutations was only about 5% both in Asian and non-Asian, [10] which made it difficult to undergo a further investigation by big scale randomized clinical trials.

Even though, further clarification of this issue was still necessary and important to find more treatment options for LSCC patients. Preliminarily literature search had found several studies published dealing with the treatment of EGFR-TKIs in EGFR mutated LSCC patients. In this study, we performed a pooled analysis to evaluate the efficacy of EGFR-TKIs in LSCC patients with EGFR mutations.

## RESULTS

### Literatures search and patient allocation

**Figure 1:**
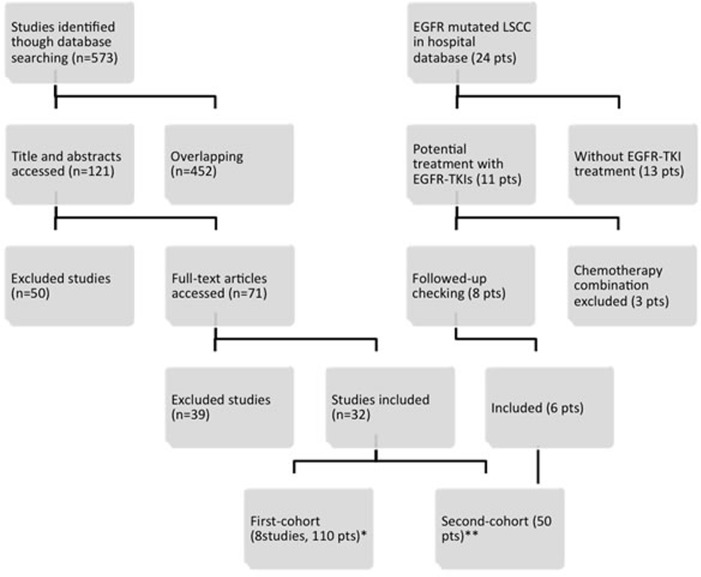
Flow diagram of literature search and eligibility Note: *EGFR mutated LSCC patients with grouped data were assigned as the first-cohort. ** EGFR mutated LSCC patients with individual data and patients from the Shanghai Chest Hospital database were allocated as the second-cohort.

A total of 71 potential records remained enrolled for full-text assessment after screened from the manual search. Of these, 32 studies were eligible for subsequent pooled analysis (Figure 1). One hundred and ten EGFR mutated LSCC patients with grouped data in the eight studies were assigned as the first-cohort (Table 1). Another forty-four EGFR mutated LSCC patients in 24 studies with individual data and six patients from the Shanghai Chest Hospital database were allocated as the second-cohort (Supplemental Table 1). The flow diagram of inclusion was showed in the Figure 1.

**Table 1:** The grouped data extracted from eight studies in the first-cohort

Reference	Country	EGFR mutation status	Gefitinib /Erlotinib /Icotinib	Response	ORR	DCR	median PFS 95%CI	*P* value
Fang2013	China	Yes, *n* = 15	NA	4PR, 6SD, 5PD	26.70%	66.70%	3.9(1.5-6.3)	0.19
		No, *n* = 48	NA	1PR, 19 SD, 28PD	2.10%	41.70%	1.9(0.7-3.2)	
Fiala2013	Czech	Yes, *n* = 16	11/5	NA	NA	NA	2.9(2.33-3.47*)	0.425
		No, *n* = 163	80/83	NA	NA	NA	1.9(1.75-2.05*)	
Hata2013	Japan	Yes, *n* = 20	18/2	1CR, 4PR, 6SD, 7PD,2NA	25.00%	50.00%	1.4(0.7-5.8)	0.1734
		No, *n* = 33	1/32	0CR, 3PR, 11SD, 8PD, 1NA	9.10%	42.40%	1.8(1.0-2.4)	
Park2009	Korean	Yes, *n* = 3	3/0	3OR	100%	100%	5.8	0.07
		No, *n* = 17	17/0	1OR	6.00%	NA	2.4	
Song2013	China	Yes, *n* = 4	NA	3OR	75.00%	NA	7.0(4.88-10.93*)	<0.001
		No, *n* = 70	NA	4OR	5.71%	NA	1.93	
Song2015	China	Yes, *n* = 4	NA	NA	NA	NA	8.0(4.44-11.56)	0.235
		No, *n* = 70	NA	NA	NA	NA	1.53(1.20-1.86)	
Xu2015	China	Yes, *n* = 22	7/11/4	7PR, 11SD, 4PD	31.80%	81.80%	3.94(2.73-5.15)	0.004
		No, *n* = 27	8/13/6	15SD, 12PD	0%	51.60%	1.94(0.89-2.99)	
Xu2016	China	Yes, *n* = 26	NA	8PR, 11SD,7PD	30.77%	73.08%	3.98(3.32-4.63)	NA

Abbreviations: ORR, object response rate; DCR, disease control rate; median PFS, median Progression-free survival; 95% CI, 95% confidence interval; CR, complete response; PR, partial response; OR, object response (CR or PR); SD, stable disease; PD, progression disease; NA, not savailable;

Note: * The 95% CIs were calculated by the Kaplan-Meier method via the data got from the survival curves.

### Characteristics of the EGFR mutated LSCC patients in the first-cohort

In the first-cohort, data was extracted in eight retrospective studies (Table 1), [11–18] including a total of 110 LSCC patients with EGFR mutation and 428 LSCC patients with EGFR wild type. Seven (87.5%) of the studies were from East Asia, another one from the Europe. The objective response rate (ORR), disease control rate (DCR), median progression-free survival (PFS) and median overall survival (OS) were available in six, four, eight and five studies. Seven studies contained LSCC patients of the EGFR mutation group and EGFR wild type group.

### EGFR mutation status in the first-cohort and the response of EGFR-TKIs

In the first-cohort, six studies reported the EGFR mutation status in a total of 86 LSCC patients with EGFR mutation. Fifty of them were exon 19 deletion, thirty-four were exon 21 L858R, and two were other types. Patients were treated with erlotinib, gefitinib and icotinib, as recorded in the publications. The response, ORR, DCR, and median PFS and the company 95% CI were extracted and shown in Table 1.

### Characteristics of the EGFR mutated LSCC patients in the second-cohort

In the second-cohort (Supplemental Table 1), there were fifty LSCC patients harboring EGFR mutations. [19–42] Most of these patients were form East Asia. The median age was 63 years, ranging from 29 to 80 years old. Thirty-four and eleven patients were male and female, with the gender of other five patients unknown. The number of smoker and non-smoker were 21 and 22, with 7 unknown. The characteristics were shown in Table 2.

**Table 2:** The characteristics of LSCC with EGFR mutations in the second-cohort

Characteristics	LSCC (*n*=50)
**Age**	
≤65	18
>65	9
NA	23
Median	63(29-80)
**Sex**	
Male	34
Female	11
NA	5
**ECOG PS**	
0	2
1	9
2	0
3	3
4	1
NA	35
**Smoke**	
YES	22
NO	21
NA	7
**TKI**	
Gefitinib	32
Erlotinib	17
Icotinib	1
**Mutation**	
Exon 19 del	19
Exon 21 L858R	13
19del+21L858R	2
G719S	1
Others	15
**Treatment sequence**	
1	8
2	12
≥3	5
NA	25
**Response**	
CR	1
PR	15
SD	18
PD	12
NE	4

Abbreviation: LSCC, lung squamous cell carcinoma; TKI, tyrosine kinase inhibitor; NA, not available; CR, complete response; PR, partial response; SD, stable disease; PD, progression disease; NE, no evaluable.

### EGFR mutation status and the response of EGFR-TKIs in the second-cohort

**Figure 2:**
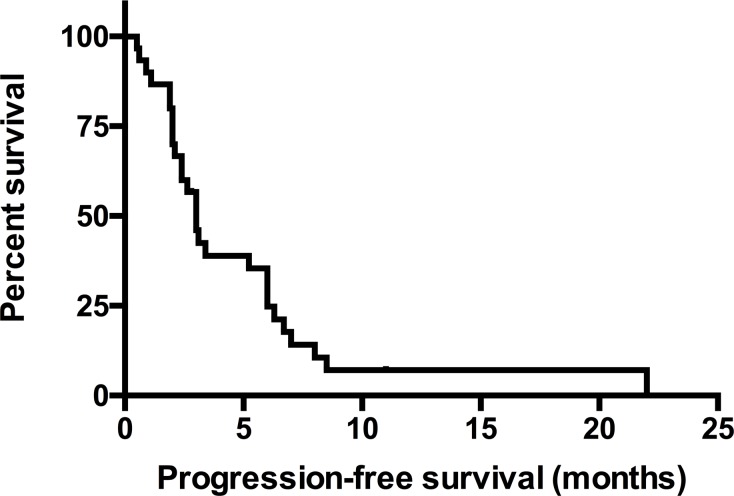
The PFS of EGFR mutated LSCC patients in the second-cohort The median PFS was 3.0 months (*n* = 30, 95% CI, 2.525–3.425 months). Abbreviations: LSCC, lung squamous cell carcinoma; PFS, progression-free survival.

In the second-cohort, EGFR mutation were defined as exon 19 deletion (*n* = 19), exon 21 L858R (*n* = 13), other mutation type were exon18 G719S, Y727H, L692P, E711K, A702S, G721A, exon 20 A763V, N826S, A859T, Q787Q, V843I, K860E, E709K, co-mutation of exon 18 E709K+ exon 21 L858R, exon 19 del + exon 21 L858R, exon 20 T790M + exon 21 L858R, exon 21 L838P + E868G. Patients were treated with gefitinib (*n* = 32), erlotinib (*n* = 17) or icotinib (*n* = 1). The ORR, DCR and median PFS were 34.78% (16/46), 73.91%(34/46), 3.0 months (Figure 2, *n* = 30, 95% CI, 2.525–3.425 months) in LSCC patients with EGFR mutation.

### Combined ORR, DCR and PFS of the first-cohort and second-cohort

**Figure 3:**
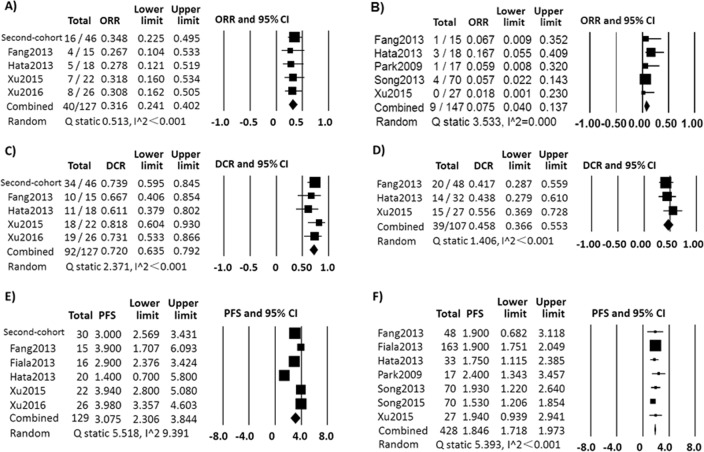
The combined results of first-cohort and second-cohort ORR in LSCC patients with EGFR mutation **A**. and with EGFR wild type **B**. treated with EGFR-TKI; DCR in LSCC patients with EGFR mutation **C**. and with EGFR wild type **D**. treated with EGFR-TKI; Median PFS in LSCC patients with EGFR mutation **E**. and with EGFR wild type **F**. treated with EGFR-TKI.

Combined ORR, DCR and PFS were calculated (Figure 3) without engaging the data contained less than five patients in any group. The combined ORR was 31.6% (*n* = 127, 95% CI, 24.1%∼40.2%; random-effect, Q statistic = 0.513, I^2^ < 0.001) in LSCC with EGFR mutation *versus* 7.5% (*n* = 147, 95% CI, 4.0%∼13.7%, random-effect, Q statistic = 3.533, I^2^ = 0.000) in LSCC with EGFR wild type. The combined DCR were 72.0% (n = 127, 95% CI, 63.5%∼79.2%, random-effect, Q statistic = 2.371, I^2^ < 0.001) in LSCC with EGFR mutation *versus* 45.8% (*n* = 107, 95% CI, 36.6%∼55.3%, random-effect, Q statistic = 1.406, I^2^ < 0.001) in LSCC with EGFR wild type. The combined median PFS was 3.08 months (*n* = 129, 95% CI, 2.31-3.84 months, random-effect, Q statistic = 5.518, I^2^ = 9.391) in LSCC with EGFR mutation *versus* 1.85 months in wild type (*n* = 428, 95% CI, 1.72-1.97 months, fix-effect, Q statistic = 5.393, I^2^ < 0.001).

## DISCUSSION

In 2014, Ameratunga et al had underwent a meta analysis on the efficacy of EGFR-TKIs to LSCC, which reveled that EGFR-TKIs had a modest therapeutic effect compared to placebo in unselected LSCC patients.[8] Then, the phase III trial Lux-Lung 8 had shown hat afatinib *versus* erlotinib as second-line treatment of patients with EGFR mutation unselected advanced LSCC had significant improvements in PFS.[7] However, these trials did not answer that whether EGFR-TKI had a better efficacy in EGFR mutated LSCC patients compared with EGFR wild LSCC patients.

This pooled analysis is by far the most updated and comprehensive analysis of EGFR-TKIs for EGFR mutated LSCC to answer this question. Takehito et al 2010 had revealed that the EGFR-TKI, gefitinib was less effective in non-adenocarcinoma NSCLC with EGFR mutation than lung adenocarcinoma (LADC) harboring EGFR mutation.[45] Our study found that EGFR mutated LSCC patients had prior response to EGFR-TKIs than EGFR wild type LSCC patients, with higher object response rate (31.6% *vs*. 11.7%), disease control rate (72.0% *vs*. 42.8%) and longer progression-free survival (3.08 *vs*. 1.85 months). This modest priority highlighted that EGFR-TKI might be a better option for EGFR mutated LSCC patients than that with EGFR wild type. However, the median PFS was shorter than that in EGFR mutated LADC.

The mechanism of lower response of EGFR-TKI treatment in LSCC patients with EGFR mutation compared to LADC patients harboring EGFR mutation is under reveling. Lee et al had found that high EGFR gene copy number could be as predictive markers for EGFR-TKI in patients with advanced squamous cell lung carcinoma.[46] Another answer was the genomic variety and complexity of LSCC. The Cancer Genome Atlas Research Network had identified the potential therapeutic gene or pathway alteration of squamous cell lung cancers, revealed that the genomic complexities were more common compared with LADC. [47, 48] Moreover, about half of all patients with LSCC carried multiple gene aberrances and 69% of the alternation was in the PI3K/ RTK/RAS signaling pathway, which might affect the efficacy of EGFR-TKI.[47] Zhijie Wang et al reported that the resistance of LSCC harboring EGFR mutation to EGFR-TKI was due to the activation of BMP-BMPR-Smad1/5-p70S6K, [49] and the combination of EGFR-TKI with inhibitors of BMP receptors signaling pathway overcame the resistance. Besides, mutation-independent mechanisms likely also contribute to the observed efficacy of EGFR-TKI therapy.[50]

Recently, the immune therapy targeting in PD-1/PD-L1 pathway had shown its priority than chemotherapy in LADC [51] and LSCC [52, 53]. However, recent pooled analysis found that EGFR mutations and ALK rearrangements were associated with low response rates to PD-1 pathway blockade in NSCLC (mainly in lung adenocarcinoma).[54] This retrospective analysis suggests that the immune therapy of PD-1 pathway blockade may not work so well in EGFR mutated NSCLC patients and EGFR mutation targeted therapy by EGFR TKI in EGFR mutated patients might be a very important selection. However, whether it is the same in EGFR mutated LSCC patients remains unknown.

Our studies had limitations. Firstly, this pooled analysis was a retrospective nature. Secondly, the very infrequency of EGFR mutation in LSCC patients had made the discovery so difficult that there were only scattered studies and reports found from the literature search, published in lower impact journals, lacking of crucial clinical information. Even we tried attempting to collect more and better data, the reliability of combining these outcomes was questionable. All of the limitations would affect the likely results. Therefore, further prospective randomized control trials are warranted to make a validation and certification of the results.

In conclusion, our study had reveled that first generation EGFR-TKIs had a modest better efficacy for LSCC patients with EGFR mutation than EGFR wild type LSCC patients, and might be a selective option for those patients with EGFR mutation. Larger prospective randomized control trials are warranted to confirm the efficacy of EGFR-TKIs targeted therapy in EGFR-mutated LSCC patients.

## MATERIALS AND METHODS

### Patients

Advanced stage (IIIB/IV) LSCC patients with EGFR mutations treated with EGFR-TKIs were identified in the database of Shanghai Chest Hospital from July 2014 to August 2016, or extracted from the publications searched from the online medicine databases.

### Data sources and search strategy

First, we systematically searched seven online databases including EMBASE, Medline (Ovid SP), Web of Science, Cochrane library, PubMed, ASCO meeting abstract and Google Scholar before August 2016. The search strategy included keywords and MeSH terms related to therapy using EGFR-TKIs in lung cancer and screened them for eligibility. Second, we identified the patients with EGFR mutated LSCC at the Shanghai Chest Hospital from July 2014 to August 2016 as Supplemental Table 1.

### Data extraction

In the hospital database and searched publications, we extracted the baseline clinical characteristics included age at diagnosis, sex, tumor histology, EGFR mutation status, performance status (PS), smoking history and prior treatment regions. Clinical treatment regimen of EGFR-TKI and response outcome was extracted or accessed. Tumor response was assessed by RECIST (version 1.1), WHO criteria and ECOG criteria in the original publications. The same terms from EGFR wild type LSCC patients were also yielded as controls. Data were recorded as individual type and grouped type, which decided the patients into different cohort for analysis.

### Statistic methods

Firstly, in the eight studies with grouped data extracted, which had its outcome of ORR, DCR and median PFS (see Table 1) in each studies, [11–17, 55] were allocated as the first-cohort.

Secondly, the searched patients with the terms extracted individually [19–42] were put as the second-cohort. The ORR and DCR were accounted. The median PFS were generated by the Kaplan–Meier method directly. These statistical analyses were performed using IBM SPSS Statistics version 22.0 for Mac OS.

Thirdly, the ORR, DCR and median PFS of the first-cohort were pooled with those in the second-cohort using Comprehensive Meta Analysis software (Version 3.13) by one-group dichotomous (for ORR and DCR) or continuous analysis functions (for median PFS) (Figure 3). Homogeneity was tested by the Q statistic (significance level at *P* > 0.10) and the I^2^ statistic (significant heterogeneity, I^2^>50%). If there was no significant heterogeneity in the groups, the fixed-effects model (Mantel–Haenszel method) was used. Otherwise, the random-effects model (DerSimonian and Laird method) was used. All tests were two-sided and P≥0.05 were considered significant.

## SUPPLEMENTARY MATERIALS TABLE


