# Supplementary material for: Efficacy of epidermal growth factor receptor (EGFR)-tyrosine kinase inhibitors (TKIs) in targeted therapy of lung squamous cell carcinoma patients with EGFR mutation: a pooled analysis
Source: Oncotarget. 2017 Feb 25;8(32):53675–83. doi: 10.18632/oncotarget.15726 (PMC5581140; doi:10.18632/oncotarget.15726)
Supplement: Supplementary file 1 [file oncotarget-08-53675-s001.doc]

**Supplement Data**

Supplementary Table 1.The basic information of 50 EGFR mutated LSCC patients in the second-cohort.

| Reference | Country | Age | Sex | Smoke | PS | Exon | Mutation | Line | TKI | Response | PFS | Censor |
| --- | --- | --- | --- | --- | --- | --- | --- | --- | --- | --- | --- | --- |
| Ann Oncol. 2005 Aug;16(8):1334-42. | China | NA | M | NO | NA | 19+21 | 19del, L858R | NA | Gefitinib | SD | 3.1 | 1 |
| Ann Oncol. 2008;19(4):739-45. | Switzerland | NA | NA | NA | NA | 21 | L858R | 1 | Gefitinib | NE | NA | NA |
| Br J Cancer. 2006 Oct 23;95(8):998-1004. | Japan | 63 | F | Yes | NA | 19 | 19 del | 1 | Gefitinib | PD | 0.5 | 1 |
| Br J Cancer. 2007 Dec 3;97(11):1560-6. | Greece | NA | M | NO | NA | 19 | 19 del | NA | Gefitinib | SD | NA | NA |
| Br J Cancer. 2007 Dec 3;97(11):1560-6. | Greece | NA | M | NO | NA | 19 | 19 del | NA | Gefitinib | SD | NA | NA |
| Br J Cancer. 2007 Dec 3;97(11):1560-6. | Greece | NA | F | NO | NA | 21 | L858R + E709K | NA | Gefitinib | PR | NA | NA |
| Br J Cancer. 2007 Dec 3;97(11):1560-6. | Greece | NA | M | Yes | NA | 18 | E711K | NA | Gefitinib | PD | NA | NA |
| Br J Cancer. 2007 Dec 3;97(11):1560-6. | Greece | NA | M | Yes | NA | 21 | K860E | NA | Gefitinib | PD | NA | NA |
| Br J Cancer. 2007 Dec 3;97(11):1560-6. | Greece | NA | M | Yes | NA | 21 | V843I | NA | Gefitinib | SD | NA | NA |
| Br J Cancer. 2007 Dec 3;97(11):1560-6. | Greece | NA | M | Yes | NA | 18 | L692P | NA | Gefitinib | PD | NA | NA |
| Br J Cancer. 2007 Dec 3;97(11):1560-6. | Greece | NA | M | Yes | NA | 18 | Y727H | NA | Gefitinib | SD | NA | NA |
| Br J Cancer. 2008 Mar 11;98(5):907-14 | Japan | NA | F | NO | NA | 19+21 | 19del, L858R | NA | Gefitinib | PR | NA | NA |
| Cancer. 2007;109(9):1836-44. | Japan | NA | M | Yes | NA | 19 | 19 del | NA | Gefitinib | PD | 0.9 | 1 |
| Cancer. 2007;109(9):1836-44. | Japan | NA | M | Yes | NA | 19 | 19 del | NA | Gefitinib | SD | 1.1 | 1 |
| Case Rep Med. 2013;2013:874836. | US | 39 | M | NO | NA | 19 | 19 del | 2 | Erlotinib | PR | 3.0 | 1 |
| Case Rep Oncol. 2013 May 14;6(2):263-8. | Japan | 64 | M | Yes | NA | 21 | L858R | 2 | Erlotinib | PR | 22.0 | 1 |
| Clin Cancer Res. 2005 Mar 15;11(6):2244-51. | Korea | 54 | F | NO | NA | 19 | 19 del | NA | Gefitinib | PR | NA | NA |
| Clin Cancer Res. 2005 Mar 15;11(6):2244-51. | China | 63 | M | NA | NA | 19 | 19 del | NA | Gefitinib | SD | NA | NA |
| Clin Cancer Res. 2005 Mar 15;11(6):2244-51. | China | 70 | M | NA | NA | 21 | Q787Q | NA | Gefitinib | PD | NA | NA |
| Clin Cancer Res. 2005 May 15;11(10):3750-7. | Taiwan | 80 | F | NO | 3 | 20 | A763V | 1 | Gefitinib | PD | 1.9 | 1 |
| Clin Cancer Res. 2005 May 15;11(10):3750-7. | Taiwan | 29 | M | Yes | 3 | 21 | L858R | 3 | Gefitinib | PD | 2.0 | 1 |
| Clin Cancer Res. 2005 May 15;11(10):3750-7. | Taiwan | 56 | M | NO | 1 | 19 | 19 del | 3 | Gefitinib | SD | 2.4 | 1 |
| Clin Cancer Res. 2005 May 15;11(10):3750-7. | Taiwan | 57 | M | Yes | 1 | 21 | N826S | 2 | Gefitinib | SD | 6.7 | 1 |
| Clin Cancer Res. 2008 Apr 1;14(7):2088-94 | US | NA | M | NO | NA | 19 | exon19，NA | 2 | Erlotinib | PR | NA | NA |
| Int J Cancer. 2007 Mar 15;120(6):1239-47. | Japan | NA | M | NA | NA | 18 | G719S | NA | Gefitinib | SD | 6.3 | 1 |
| Intern Med. 2012;51(6):659-61 | Japan | 56 | M | Yes | 4 | 19 | 19 del | 1 | Gefitinib | PR | 2.0 | 1 |
| J Cancer Res Clin Oncol. 2009 Jun;135(6):771-82. | China | NA | F | NO | NA | 18 | G721A | NA | Gefitinib | CR | 7.0 | 1 |
| J Cancer Res Clin Oncol. 2009 Jun;135(6):771-82. | China | NA | F | NO | NA | 18 | A702S | NA | Gefitinib | PR | 8.5 | 1 |
| J Cancer Res Clin Oncol. 2009 Jun;135(6):771-82. | China | NA | M | NO | NA | 19 | 19 del | NA | Gefitinib | SD | 11.0 | 0 |
| J Clin Oncol. 2005;23(11):2493-501. | Korea | NA | F | NO | NA | 21 | A859T | NA | Gefitinib | PD | 2.0 | 1 |
| J Clin Oncol. 2007;25(7):760-6. | US | 73 | M | Yes | NA | 21 | L858R | 1 | Erlotinib | SD | 3.4 | 1 |
| Jpn J Clin Oncol. 2011 Dec;41(12):1366-72. | Japan | NA | NA | NO | NA | 19 | 19 del | NA | Erlotinib | NE | NA | NA |
| Jpn J Clin Oncol. 2011 Dec;41(12):1366-72. | Japan | NA | NA | NO | NA | 19 | 19 del | NA | Erlotinib | NE | NA | NA |
| Lung Cancer. 2006;53(3):311-22. | Taiwan | 59 | F | NO | NA | 21 | L838P/E868G | 2 | Gefitinib | PD | NA | NA |
| Lung Cancer. 2006;53(3):311-22. | Taiwan | 75 | M | Yes | NA | 21 | L858R | 3 | Gefitinib | SD | NA | NA |
| Lung Cancer. 2011;73(3):379-80. | Japan | 61 | M | Yes | 3 | 19 | 19 del | 1 | Gefitinib | PR | 8.0 | 1 |
| Lung Cancer. 2012;77(1):121-7. | Korea | 65 | F | NO | NA | 19 | 19 del | 2 | Gefitinib | NE | 5.23 | 1 |
| Lung Cancer. 2012;77(1):128-33. | Taiwan | NA | NA | NA | NA | 21+20 | T790M, L858R | NA | Erlotinib | PD | NA | NA |
| Lung Cancer. 2012;77(1):128-33. | Taiwan | NA | NA | NA | NA | 19+21 | 19del, L858R | NA | Erlotinib | PR | NA | NA |
| Mol Cancer Ther. 2012 Nov;11(11):2535-40. | US | 58 | M | NO | NA | 21 | L858R | 1 | Erlotinib | PR | 1.9 | 1 |
| Mol Cancer Ther. 2012 Nov;11(11):2535-40. | US | 68 | M | NO | NA | 21 | L858R | NA | Erlotinib | PR | 2.8 | 0 |
| Oncology. 2014;86(2):86-93. | Japan | 75 | M | Yes | 0 | 19 | 19 del | 3 | Erlotinib | PD | 0.6 | 1 |
| Oncology. 2014;86(2):86-93. | Japan | 54 | M | Yes | 0 | 21 | L858R | 3 | Erlotinib | SD | 2.1 | 1 |
| Oncology. 2014;86(2):86-93. | Japan | 72 | M | Yes | 1 | 19 | 19 del | 2 | Erlotinib | SD | 2.4 | 1 |
| Shanghai Chest Hospital | China | 63 | M | Yes | 1 | 21 | L858R | 2 | Icotinib | SD | 2.63 | 1 |
| Shanghai Chest Hospital | China | 39 | M | NA | 1 | 21 | L858R | 2 | Erlotinib | SD | 3.0 | 1 |
| Shanghai Chest Hospital | China | 69 | F | NO | 1 | 21 | L858R | 2 | Erlotinib | SD | 3 | 1 |
| Shanghai Chest Hospital | China | 47 | M | Yes | 1 | 19 | 19del | 1 | Erlotinib | PR | 6 | 1 |
| Shanghai Chest Hospital | China | 64 | M | Yes | 1 | 21 | L858R | 2 | Erlotinib | PR | 6 | 1 |
| Shanghai Chest Hospital | China | 74 | M | NO | 1 | 21 | L858R | 2 | Gefitinib | PR | 6 | 1 |

Abbreviations: PS, ECOG performance score; M, male; F, female; NA, no available; NE, no evaluable.
